# A new lymph node infection model for *Streptococcus suis* serotype 2 in pigs

**DOI:** 10.1186/s13567-025-01616-7

**Published:** 2025-10-02

**Authors:** Annika Katharina Breitfelder, Tatjana Sattler, Johannes Kauffold, Sarah Pfetzing, Anna Majcher, Reiner Ulrich, Uwe Müller, Sophie Öhlmann, Karoline Rieckmann, Christoph Georg Baums

**Affiliations:** 1 Institute of Bacteriology and Mycology, Centre for Infectious Diseases, Faculty of Veterinary Medicine, Leipzig University, Leipzig, Germany; 2 Clinic for Ruminants and Swine, Faculty of Veterinary Medicine, Leipzig University, Leipzig, Germany; 3 Institute of Veterinary Pathology, Faculty of Veterinary Medicine, Leipzig University, Leipzig, Germany; 4 Institute of Immunology, Centre for Infectious Diseases, Faculty of Veterinary Medicine, BBZ, Leipzig University, Leipzig, Germany

**Keywords:** *Streptococcussuis*, lymph node infection model, bacterial dissemination, *Lymphonoduscervicalissuperficialisdorsalis*, immunohistochemistry, Iba-1, mononuclear phagocytic system

## Abstract

*Streptococcus suis* (*S*. *suis*) is an important invasive porcine pathogen. Here, we describe a new lymph node infection model where 7 × 10^3^ CFU *S*. *suis* serotype 2 were injected in the left cervical lymph node. In total, seven out of eight 5-week-old piglets developed clinical signs of disease. In all infected pigs, *S*. *suis* was reisolated from the infected lymph node and one or more additional sites. Immunohistochemically, *S*. *suis* antigen was located predominantly in the sinus. The results suggest that *S*. *suis* easily disseminates after lymph node infection and that the interaction with cells of the mononuclear phagocytic system, primarily in the lymph node sinus, plays an important role in host cell–pathogen interaction.

**Supplementary Information:**

The online version contains supplementary material available at 10.1186/s13567-025-01616-7.

## Introduction, methods, and results

*Streptococcus suis* (*S*. *suis*) is one of the most important porcine pathogens worldwide, causing severe diseases, such as meningitis, polyarthritis, septicemia, or sudden death, mostly in piglets between 4 and 10 weeks of age [1, 2]. Additionally, *S*. *suis* very successfully colonizes the tonsils and mucosal surfaces of the upper respiratory tract of up to 100% of pigs of all ages without causing disease [3].

Lymph nodes are an important immune check-point, where peripheral antigens or pathogens come in contact with antigen-presenting cells and macrophages [4]. Additionally, B cell follicles are an important site of antigen-induced B cell activation and differentiation [5].

In contrast to most other mammals, such as mice and humans, the porcine lymph nodes present an inverted structure, with a central cortex and peripheral medulla and centrifugal lymph flow from the center to the periphery [6, 7]. Lymphocytes enter directly through high endothelial venules and leave the lymph nodes directly into the blood via efferent blood vessels instead of via efferent lymphatics [6, 8].

Lymphatic metastasis, the spread of a pathogenic agent via the lymphatic system, is mainly acknowledged for bacteria which survive inside phagocytic cells, such as *Salmonella enterica* [9]. However, newer research challenges the dogma that severe bloodstream and systemic infections arise after bacterial invasion of blood vessels at the primary infection site. Instead, it suggests that lymphatic spread and the interaction between pathogen and the hosts immune system within the lymph node is of greater importance than previously assumed [10].

*S*. *suis* has evolved several mechanisms to interact with the pig’s immune system. The polysaccharide capsule as critical virulence factor determines the classification into different serotypes (cps) and protects against phagocytosis [11, 12]. In addition, *S*. *suis* can be found in association with blood leukocytes [13, 14] and expresses Ide_*Ssuis*_, a highly specific protease able to cleave soluble porcine IgM as well as the IgM B cell receptor on blood and lymph node B cells [15, 16]. *S*. *suis* was frequently detected in different lymph nodes of naturally infected pigs showing signs of joint disease, meningitis or sudden death [17]. Additionally, *S*. *suis* causes more severe lymph node lesions in pigs primarily infected with the porcine reproductive and respiratory syndrome virus (PRRSV) [18]. Taken together, these findings suggest that interaction of *S*. *suis* with immune cells in the lymph node might contribute to dissemination and pathogenesis. Thus, we aimed to establish a new infection model to investigate the interaction between *S*. *suis* and the porcine immune system in the lymph node.

### Bacterial strain and growth conditions

*S*. *suis* strain 10 (*epf*+*gdh*+*sly*+*mrp*+) is a well characterized sequence type 1 serotype 2 strain frequently used in animal experiments [19–21]. *S*. *suis* was grown on Columbia blood agar plates (Thermo Oxoid, Schwerte, Germany) or in Tryptic-Soy-Broth without dextrose (TSB) (Bacto, BD, Heidelberg, Germany) at 37 °C with 5% CO_2_.

### Bactericidal assay

Survival and proliferation of *S*. *suis* strain 10 in porcine blood ex vivo was investigated as described previously [16] prior to lymph node infection to determine susceptibility of the trial animals to *S*. *suis* strain 10 challenge. Blood samples were taken at the age of 5 weeks parallel to lymph node injection (see below). Briefly, 500 µL of heparinized whole blood were mixed with 5 × 10^6^ CFU *S*. *suis* strain 10 and incubated for 120 min at 37 °C. The survival factor was calculated by dividing CFU_*t*=120 min_ by CFU_*t* =0 min_.

*S*. *suis* strain 10 was able to proliferate in the blood from 9 out of 10 pigs, with survival factors ranging from 1.6 to 8.2. Only the blood of pig 5 was able to limit *S*. *suis* survival (survival factor 0.8) (Additional file 1). In total, these data suggest a high susceptibility to *S*. *suis* strain 10 infection in these animals.

### Animal experiment and clinical signs

Ten 4-week-old German Landrace piglets from a herd known to be free of *epf*+*gdh*+*sly*+*mrp*+ *S*. *suis* cps 2 strains, as well as *cps* 1, *cps* 14, *cps* 7 and *cps* 9, were transported to the Faculty of Veterinary Medicine Leipzig.

At an age of 5 weeks, 8 animals were experimentally infected in the left *Lymphonodus* (Ln.) *cervicalis superficialis dorsalis* with 7 × 10^3^ CFU of *S*. *suis* strain 10, suspended in 150 µL PBS. For that, the pigs were anaesthetized by intramuscular injection of 2 mg/kg azaperone (Stresnil ®, Elanco Deutschland GmbH, Bad Homburg, Germany, via WDT, catalog 04603) and 20 mg/kg ketamine-hydrochloride (Ursotamin ®, Serumwerk Bernburg AG, Bernburg, Germany, via WDT, catalog 08869). The skin above the left cervical lymph node was disinfected, incised and the lymph node was prepared in situ to allow for injection under visual control. Finally, the skin was sutured using non-aborsable threads (Additional file 2). The pigs received additional intramuscular buprenorphine (Bupresol ®, CP-Pharma, Burgdorf, Germany, via WDT, catalog 04928) injections for pain management (0.01 mg/kg during anesthesia for lymph node injection and 0.05 mg/kg 8 h postoperatively). Two pigs remained as control animals and did not undergo surgery. Tonsil swabs were taken during surgery to determine the *S*. *suis* carrier status and blood samples from all animals were taken in parallel to investigate the bacterial survival in blood ex vivo, respectively. *S*. *suis* was isolated from the tonsils on the basis of MALDI-TOF MS analysis and detection of *gdh* but none of the isolates generated an amplicon for *mrp*, *epf*, *sly*, *cps1* or *cps14*, *cps2* or *cps1/2*, *cps7 or cps9* in the MP-PCR assay [23]. The health status of the animals was scored every 8 h and humane end points were defined as described by Liedel et al. [22]. Group 1 (control animal #1 and challenged pigs #3–6) were sacrificed 24 h post-infection for sample collection. Group 2 (control animal #2 and challenged pigs #7–10) was further observed for clinical signs of *S*. *suis* disease until 4 days post-infection. After surgery, all pigs recovered rapidly and displayed normal body temperature, good feed intake and normal behavior without signs of pain 4 h postoperatively. Additionally, the skin wound showed good healing , with only slight inflammation of the wound edges in one out of eight pigs.

In group 1, three out of four pigs showed increased body temperature, recorded as early as 12 h post-infection. One animal displayed high-grade lameness on one limb in combination with tremor, and another one showed a reduced feed intake and behavioral depression (Table 1). In the second group, greatly elevated body temperature was recorded in all pigs (Table 1). At 48 h post-infection, pigs 7 and 8 showed additional ceased food intake, behavioral depression, high-grade lameness on one leg, and vomitus or local tremor at two consecutive time points and were therefore euthanized for animal welfare reasons. At 56 h post-infection, pig 9 reached the humane end points because of fever, ceasing food intake, behavioral depression, high-grade lameness on the right hind leg, and generalized tremor. At 70 h post-infection, the last animal (pig 10) was euthanized because of fever, ceasing food intake, apathy, low-grade lameness on the right hind leg, generalized tremor, vomitus, and kyphosis. Both uninfected control animals did not show any clinical signs of disease (Table 1).

**Table 1:** **Morbidity, mortality, and clinical signs after** ***S. suis*** **strain 10 injection in the cervical lymph node**

	Morbidity^a^	Mortality	Clinical signs				Maximum body temperature (°C)				
			CNS	Lameness	No feed intake	Unspecific^b^	< 40	40–40.2	40.3–40.5	40.5–40.9	≥ 41
Noninfected	0/2	0/2	0/2	0/2	0/2	0/2	2/2	0/2	0/2	0/2	0/2
Infected (total)	6/8	4/8	1/8	5/8	5/8	5/8	1/8	1/8	0/8	2/8	4/8
• Group 1	2/4	0/4	0/4	1/4	1/4	1/4	1/4	1/4	0/4	1/4	1/4
• Group 2	4/4	4/4	1/4	4/4	4/4	4/4	0/4	0/4	0/4	1/4	3/4

^a^ Morbidity was defined as inner body temperature > 40.2 °C.

^b^ Change in behavior, depression, or vomitus.

### Post-mortem findings

During necropsy, the following samples were collected for histological (h) and bacteriological (b) investigation: cerebrospinal fluid (CSF) (b); brain (h); brain swab (b); tarsal and carpal joints (h); tarsal and carpal joint fluid (b); peritoneal, pleural, pericardial, and bicuspidalis swabs (b); peritoneum, pleura, and pericardium (h); left heart valve (b, h); cranial lobe of the left lung (b, h); liver (b, h); spleen (b, h); tonsil (b, h); left and right *Ln. cervicalis superficialis dorsalis* (h, b); left and right *Ln. mandibularis* (b); left and right *Ln. inguinalis* (b); left and right *Ln. subiliacus* (b); left and right *Ln. popliteus* (b); *Ln. tracheobronchialis* (b); *Ln. jejunalis* (b). Isolation of the challenge strain was confirmed by MALDI-TOF MS with subsequent multiplex PCR for the detection of *mrp*, *epf*, *sly*, *arcA*, *gdh*, *cps1* or *cps14*, *cps2* or *cps1/2*, *cps7,* and *cps9* [23].

**Figure 1:**
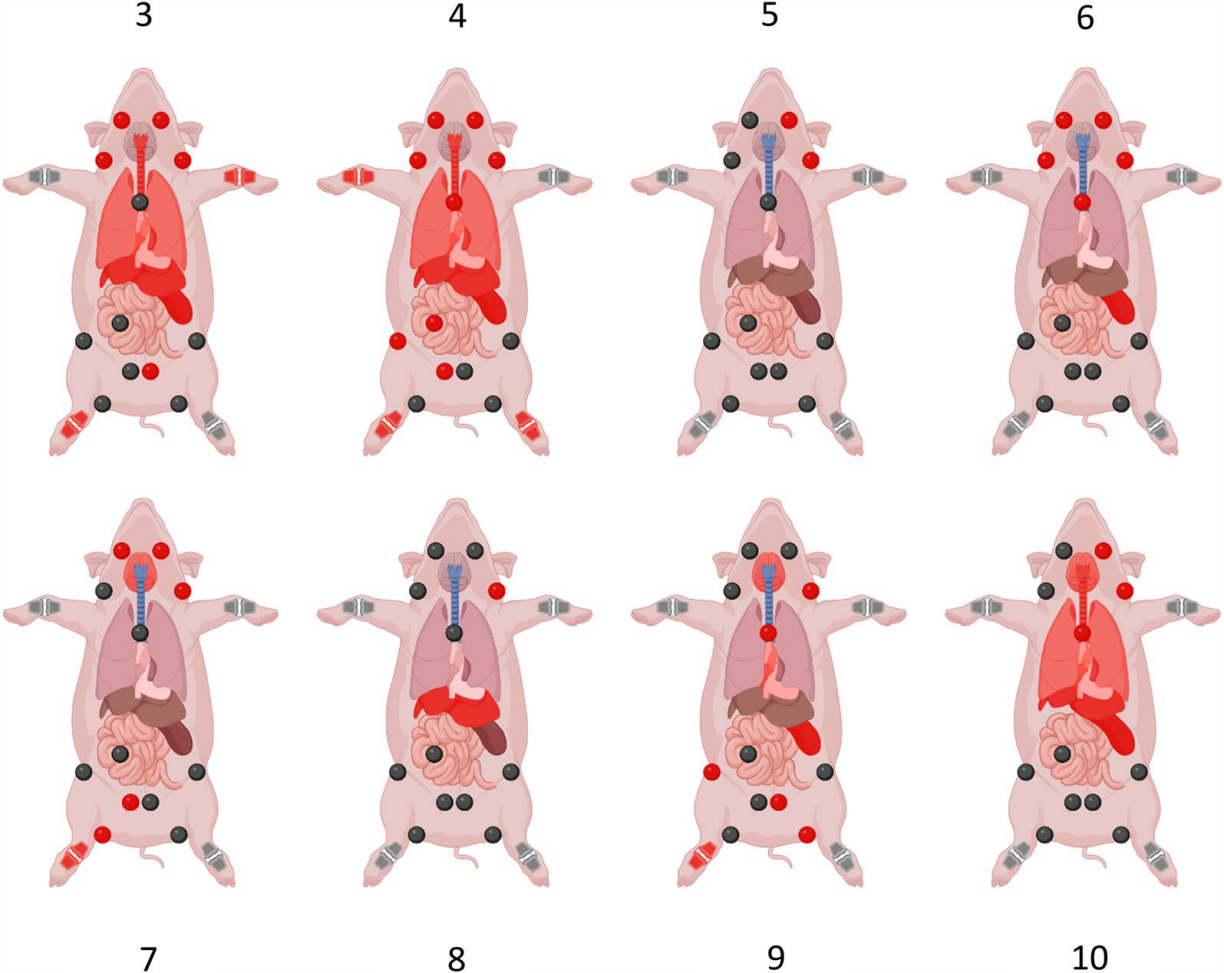
**Reisolation of the infection strain from lymph nodes, inner organs and joints from infected pigs.** Upper row: group 1, euthanized 24 h post inf. Lower row: group 2, euthanized at day 2 and 3 post-infection. Red coloring indicates reisolation of *S*. *suis* strain 10. *S*. *suis* strain 10 was not isolated from the uninfected control animals (not shown). This figure was created with Biorender.

In all infected pigs, the *S*. *suis* infection strain could be recovered from the left cervical lymph node in which it was previously injected as well as from multiple other sites (Additional files 3, 4, Figure 1). In group 1, the infection strain was not isolated from CSF or the brain in any of the animals. In pig 3 and 4, the infection strain was recovered from several inner organs, ipsilateral and contralateral lymph nodes, and multiple joints. Pig 4 also clinically presented with severe lameness in one leg. In pig 5, the infection strain was not detected in any of the inner organs or the joints. It was recovered only from the left *Ln. cervicalis* and left *Ln. mandibularis*. This pig was also the only animal to show a normal body temperature < 40 °C during the whole observation time (Table 1). Noteworthy, pig 5 was the only animal able to kill *S*. *suis* strain 10 in the blood bactericidal assay (Additional file 1). In pig 6, the challenge strain was isolated only from the spleen, but no other inner organ. Additionally, it was recovered from the *Ln. tracheobronchialis* and the lymph nodes of the head of both sides. In group 2, the infection strain was isolated from the brain, the blood, and several other sites of pigs 7, 9, and 10. In pig 10, the challenge strain was also recovered from CSF. The two pigs from which the infection strain was isolated from the tarsal joint (pig 7 and 9) also showed high-grade lameness. The *S*. *suis* infection strain was not isolated from any samples from uninfected control animals, which were housed in the same pen. Detailed information for each individual pig can be found in Additional file 5.

Histopathologically, moderate to severe acute suppurative synovialitis was observed in the joints from the pigs which also clinically presented with lameness. H.E.-stained sections of all other samples showed no lesions clearly induced by the infection (Additional file 6).

### Cervical lymph nodes

**Figure 2:**
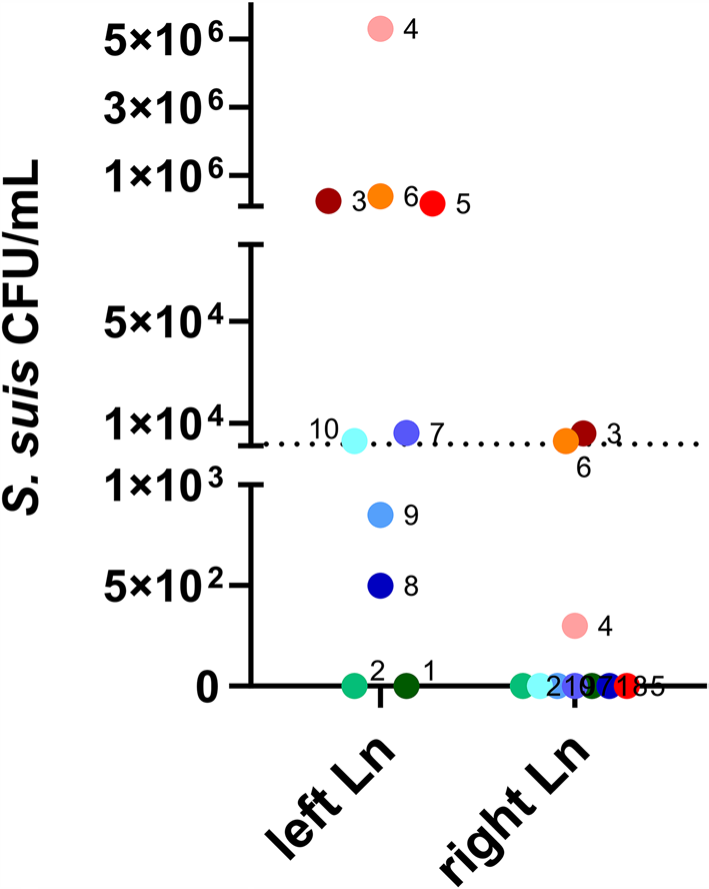
**Recovered CFU counts of *S. suis*** **from the right and left cervical lymph node**. Dotted line: CFU counts recovered after infection of the same dose (7 × 10^3^ CFU *S*. *suis*) in a cervical lymph node of a dead animal. Red: animals of group 1, blue: animals of group 2, green: uninfected control animals.

Additionally, semi-quantitative analysis of *S*. *suis* colony-forming unit (CFU) counts in the cervical lymph nodes were performed. After taking a small slice for immunohistopathological investigations, the rest of the lymph node tissue was minced. The resulting fine tissue mash was mixed with 180 µL sterile PBS. 20 µL were directly plated on blood agar plates and further 20 µL were used for a serial dilution. For comparison, the same infection dose of 7 × 10^3^ CFU *S*. *suis* strain 10 was injected in the cervical lymph nodes of a pig which was killed immediately before, the lymph node was processed in the same way and 1.7 × 10^3^ CFU/mL could be recovered (dotted line in Figure 2). In the left cervical lymph node from pigs of group 1, between 1.8 × 10^5^ and 5.3 × 10^6^ CFU/mL *S*. *suis* were recovered, indicating a prominent proliferation of the infection strain in the lymph node during the first 24 h post-infection (Figure 2). In the left cervical lymph node from pigs of group 2, *S*. *suis* strain 10 was recovered with counts between 6 × 10^3^ and 500 CFU/mL. In the contralateral cervical lymph node, *S*. *suis* was only found in pigs of group 1.

Immunohistochemistry to detect *S*. *suis* antigen was performed on cervical lymph nodes as previously described [24, 25]. A polyclonal anti-*S*. *suis* serum was raised within a previous study in a rabbit by prime-boost vaccination with a bacterin including incomplete Freuds adjuvant and *S*. *suis* strain I9841/1 (*mrp* + *epf* + *cps2* of sequence type 1) inactivated with formaldehyde. The polyclonal anti-*S*. *suis* serum has already been used successfully in immunohistochemical analysis of pigs experimentally infected with strain 10 in a previous study [24].

**Figure 3:**
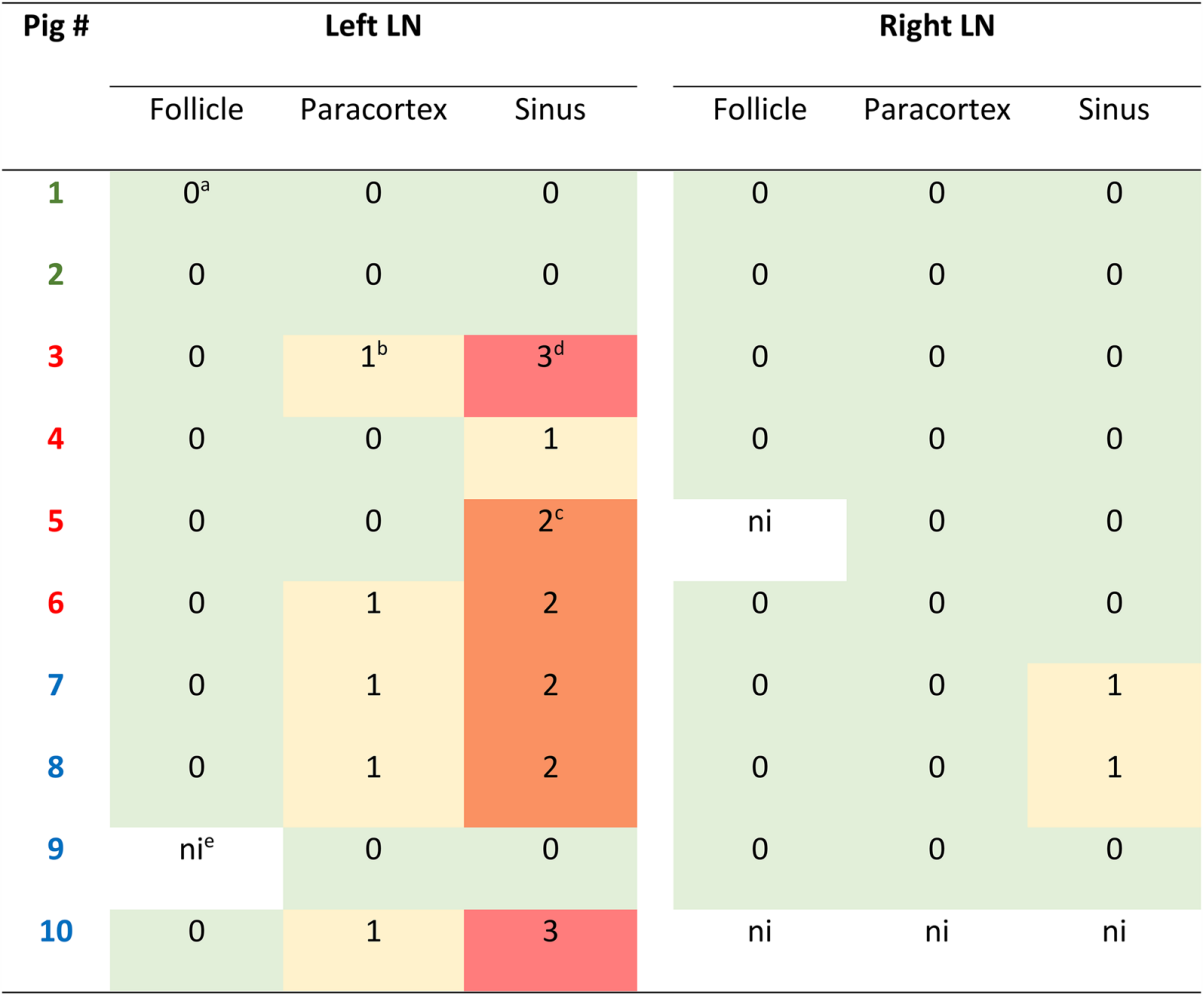
**Detection of** ***S. suis*strain 10 antigen in** ***Ln. cervicalis superficialis dorsalis***. Serial sections from the left and right cervical lymph node were immunolabeled for *S*. *suis* antigen. Pig 1,2: control group; pig 3–6: infected group 1; pig 7–10: infected group 2. ^a^0—no antigen detected; ^b^1—focal or oligofocal antigen detected; ^c^2—multifocal antigen detected; ^d^3—diffuse antigen detected; ^e^ni—not investigated.

**Figure 4:**
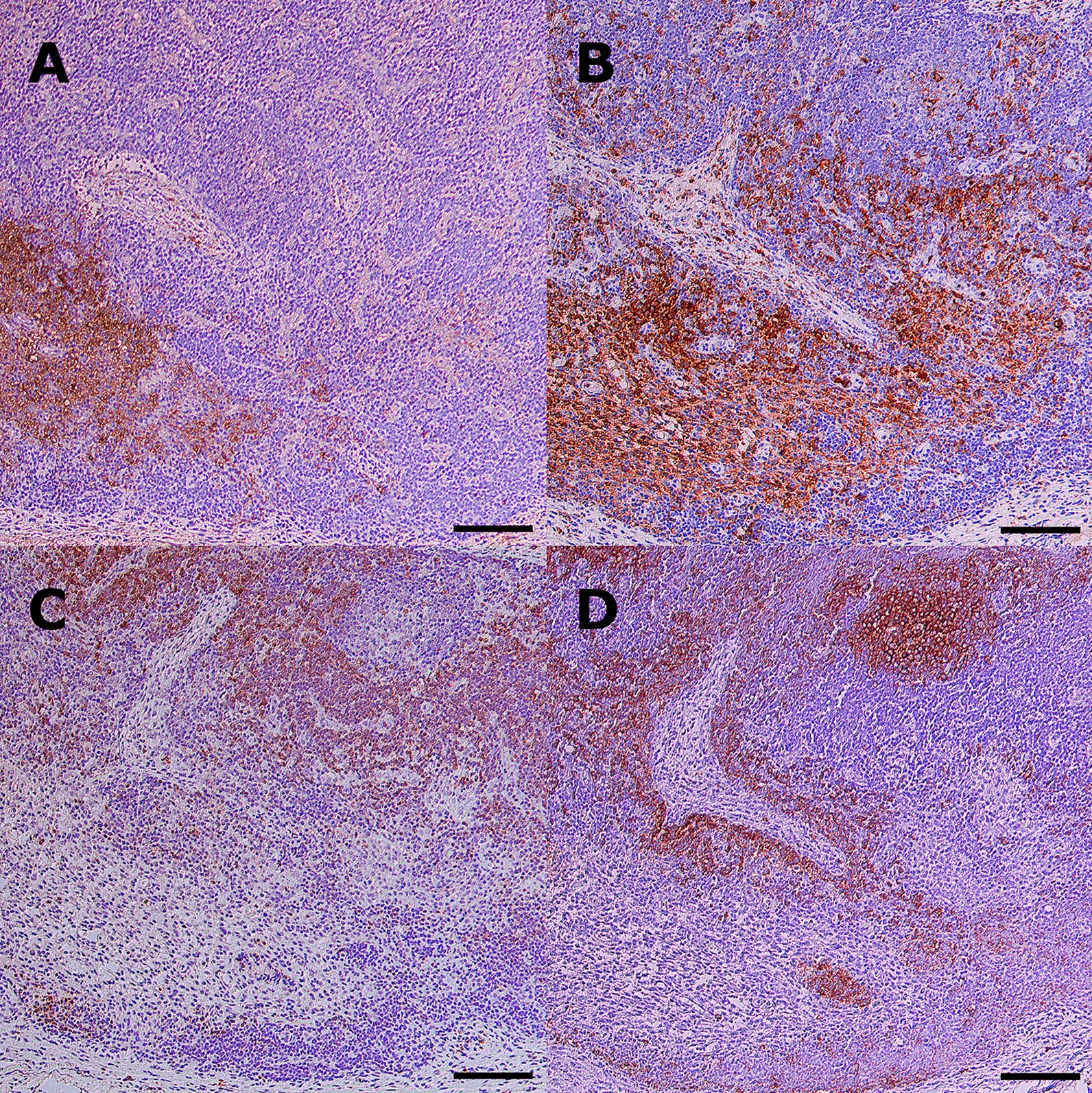
**Immunohistochemistry of a left cervical lymph node infected with *S. suis*** **cps2**. Serial sections from the left cervical lymph node of pig #6 (sacrificed 24 h post-infection) were immunolabelled for *S*. *suis* antigen (**A**), Iba-1 for monocytes and macrophages (**B**), CD3 for T-lymphocytes (**C**) and CD20 for B-lymphocytes (**D**). Avidin–biotin-peroxidase complex method with diaminobenzidine as chromogen (brown) and haematoxylin counter-stain (blue); Nomarski differential interference contrast; scale bars = 100 µm.

Primary polyclonal rabbit anti- *S*. *suis* serum (dilution 1:1600, 4 °C overnight) and secondary biotinylated goat-anti-rabbit-antibody (dilution 1:200, 30 min, #BA-1000, Vector Laboratories, CA, USA) were used to label *S*. *suis* antigen. Iba-1 was used as a marker for monocytes and macrophages, CD3 for T cells and CD20 for B cells. *S*. *suis* antigen was detected predominantly in the medullary sinuses of the left cervical lymph nodes, and to a lesser extent in the paracortical zone (Figure 3, Additional file 7). However, no antigen could be detected in the follicles. Immunolabeling of serial lymph node sections further illustrated *S*. *suis* antigen in the medullary sinuses, a location highly enriched in cells of the mononuclear phagocytic system (Figure 4). In contrast, T cells and B cells are primarily located in the paracortex or perivascular and in the follicles, respectively.

Histomorphologically, no follicular or paracortical hyperplasia was seen, suggesting that there was no prominent activation of the lymph nodes during the observation period. Mild to moderate neutrophilic infiltration was seen in all animals and mild purulent lymphadenitis was found in the left cervical lymph nodes of three pigs. However, neutrophilic infiltration was also present in pigs from the control and infection group as well as in the contralateral cervical lymph nodes, indicating that a mild to moderate degree of neutrophilic infiltration of lymph nodes might be due to some other immune response and did not result from the performed *S*. *suis* infection (Additional file 8).

## Discussion

The lymph node is a neglected tissue in *S*. *suis* research, even though results of several studies suggest that lymph nodes might play an important role during pathogenesis. For example, Bornemann et al. determined *S*. *suis* isolation frequencies in lymph nodes of naturally infected pigs showing clinical signs of *S*. *suis* disease [18]. In 38% of animals, *S*. *suis* was found in at least one of the investigated lymph nodes, and in 24.9% of animals, more than one lymph node was positive for *S*. *suis*. Additionally, *S*. *suis* can be found not only in lymph nodes of naturally infected pigs, but also after experimental infection [26]. Madsen et al. performed an aerogenous infection of pigs with a *S. suis cps*2 strain. *S. suis cps*2 antigen was detected in the tonsils of all challenged animals and also in the mandibular lymph node in the animals which developed clinical signs [27]. These results point to the tonsil as a possible route of entry with possible subsequent lymphogenic spread.

A suitable animal model focusing on lymph node infection does not exist. The majority of experimental *S*. *suis* infection is performed via the intravenous or intranasal route [28, 29]. However, intravenous infection bypasses the natural portal of entry [30]. Intranasal infection of conventional [28, 30], but not necessarily caesarian-derived, colostrum-deprived (CDCD) piglets [31], also requires mucosal irritation by acetic acid or application of an predisposing infectious agent such as PRRSV prior to *S*. *suis* application to induce disease. Therefore, we aimed to develop a porcine model of *S*. *suis* lymph node infection. While accessing the lymph node by surgery is still bypassing the first initial steps of *S*. *suis* breaching the porcine mucosal barriers, it allows for the investigation of lymphogenous spread, an important but still neglected factor of *S*. *suis* infection.

We chose the *Ln. cervicalis superficialis dorsalis* for injection of streptococci because of anatomical and practical considerations. The lymph node is located partially underneath the *Musculus omotransversarius*, surrounded by fat tissue, and in no direct proximity to important nerves or bigger blood vessels. Furthermore, it can be accessed via the outer skin and the resulting suture is in a region of reduced skin movement to facilitate healing. In addition, the tonsil is considered the natural niche for *S*. *suis* and is discussed as possible route of entry [3, 27, 32]. The *Ln. cervicalis superficialis dorsalis* acts as a major downstream lymph node to the tonsil and mandibular and submandibular lymph nodes [33, 34].

Even though we chose a very low infection dose of 7 × 10^3^ CFU compared with infection doses used for intravenous or intranasal infection (2 × 10^8^ CFU or 1.5 × 10^9^ CFU, respectively) [21, 35], we saw a very rapid onset of systemic infection. We found an early infection of the joints in group 1, and infection of the brains only at later time points in group 2. In all infected pigs, *S*. *suis* was reisolated from at least two different sites (Figure 1). Therefore, our results correspond to data in naturally infected pigs where *S*. *suis* can be detected in multiple inner organs as well as in multiple lymph nodes [18]. An infected lymph node is often considered as indication for an infection in the tributary region [36]. In the context of studies trying to establish a gastrointestinal infection in pigs, reisolation of the challenge strain from gastrointestinal lymph nodes was used to prove translocation via the gut [37, 38]. However, the results presented in this study demonstrate that as soon as a systemic infection is established, it is impossible to trace back the original route of entry on the basis of positive lymph nodes. Here, we isolated *S*. *suis* from the lung without parallel isolation from the tracheobronchial lymph node and vice versa. In one pig, we found a positive lymph node of the small intestine without previous gastrointestinal infection. Furthermore, *S*. *suis* was also isolated from joints or the brain without corresponding clinical or histopathological findings in some pigs. We speculate that this is because of the very rapid onset of infection, and that the clinical or histopathological manifestations would have been more prominent if the animals had lived longer.

In all pigs, *S*. *suis* was reisolated from the injected lymph node, with bacterial loads up to almost 1000 times greater than the infection dose. These findings demonstrate that *S*. *suis* is able to proliferate and persist in lymph nodes for several days. The role of specific immune evasion mechanism which might help this pathogen to thrive in lymph nodes for longer time periods still needs to be elucidated. Specifically, the role of IgM B cell receptor cleavage in silencing B cells and the role of binding to the surface of leukocytes in dissemination (modified trojan horse theory) might be investigated in this model. Neila-Ibáñez et al. [39] suggested that the interaction with lymph nodes could partially explain why some strains become invasive and others do not. The location of *S*. *suis* antigen mainly in the sinusoids suggests that cells of the mononuclear phagocytic system are an important host cell interacting with *S*. *suis* in lymph nodes. In the porcine lymph node, perifollicular macrophages most likely play an important role in the translocation of antigen from the sinus to the follicles and in B cell maturation [4, 6]. However, direct interaction of *S*. *suis* with B cells in lymph nodes might be limited, as suggested by the immunohistochemical findings of this study (Figure 4).

Siggins et al. investigated the extracellular lymphatic metastasis of *S. pyogenes* after intramuscular injection in a mouse model [40]. *S. pyogenes* remained extracellular (free or attached to lymphocytes) and transitioned from the local infection site through local and sequential draining lymph nodes to the blood stream and inner organs. 24 h post-infection, *S. pyogenes* was also found in the contralateral lymph nodes. In our study, as several organs and ipsilateral and contralateral lymph nodes were found positive, we cannot say whether *S*. *suis* traveled via the lymphatics or directly entered the blood stream in the infected lymph node. The location of *S*. *suis* antigen predominantly in the sinus points to a possible lymphogenous spread. On the other hand, the porcine lymph node is characterized by unique circulation, where lymphocytes from the lymph node directly re-enter the circulation via high-endothelial venules in the paracortex [8]. This could be of special importance if *S*. *suis* travels attached to the outside of lymphocytes (modified trojan horse theory) [14].

## Supplementary Information

**Additional file 1. *S. suis*** **strain 10 survival factors prior to infection.**

**Additional file 2. Surgery to inject* S. suis* in the left cervical lymph node.**

**Additional file 3. Reisolation of the infection strain from inner organs or joint fluid.**

**Additional file 4. Reisolation of the infection strain from lymph nodes.**

**Additional file 5. Detailed information to the individual pigs.**

**Additional file 6. Scoring of fibrinosuppurative lesions of piglets infected with 3 x 10**^**7**^** CFU of* S. suis* strain 10.**

**Additional file 7. Distribution of *S. suis*-antigen within the left cervical lymph node.** **A** Diffuse antigen within the sinusoids and paracortex. **B** Focal antigen within the sinusoids and paracortex. Serial sections from the left cervical lymph node were immunolabelled for *S*. *suis* antigen. Avidin-biotin-peroxidase complex method with diaminobenzidine as chomogen (brown) and haematoxylin counter-stain (blue); Nomarski differential interference contrast; scale bars = 20 µm.

**Additional file 8. Histomorphological lesions in *Ln. cervicalis* superficialis dorsalis.**

## Data Availability

The datasets used and/or analyzed during the current study are available from the corresponding author upon reasonable request.
