# Supplementary figures and images for: A new lymph node infection model for Streptococcus suis serotype 2 in pigs
Source: Vet Res. 2025 Oct 2;56:186. doi: 10.1186/s13567-025-01616-7 (PMC12490033; doi:10.1186/s13567-025-01616-7)

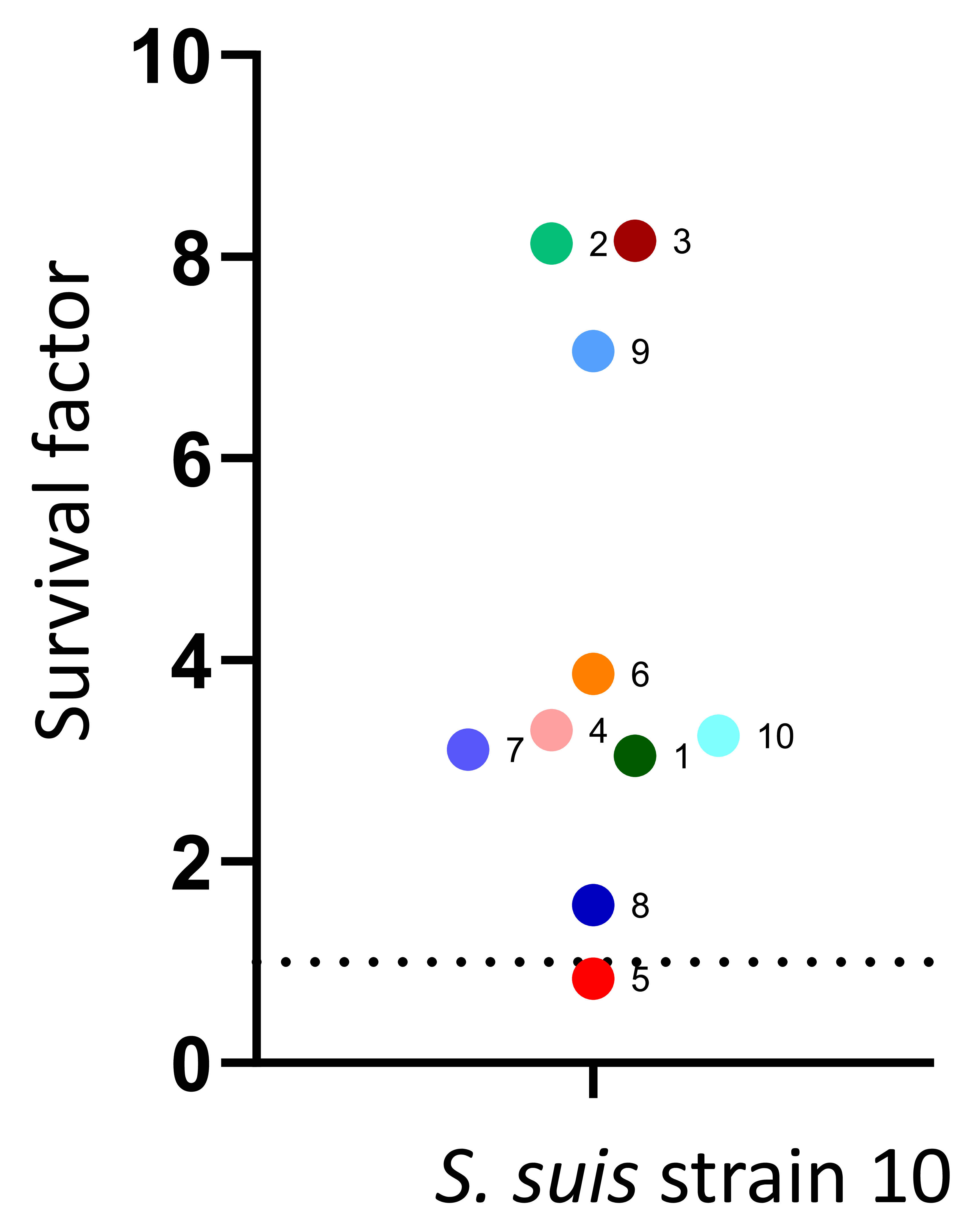

Supplement: Supplementary file 1 — Additional file 1. S. suis strain 10 survival factors prior to infection. [file 13567_2025_1616_MOESM1_ESM.tif]

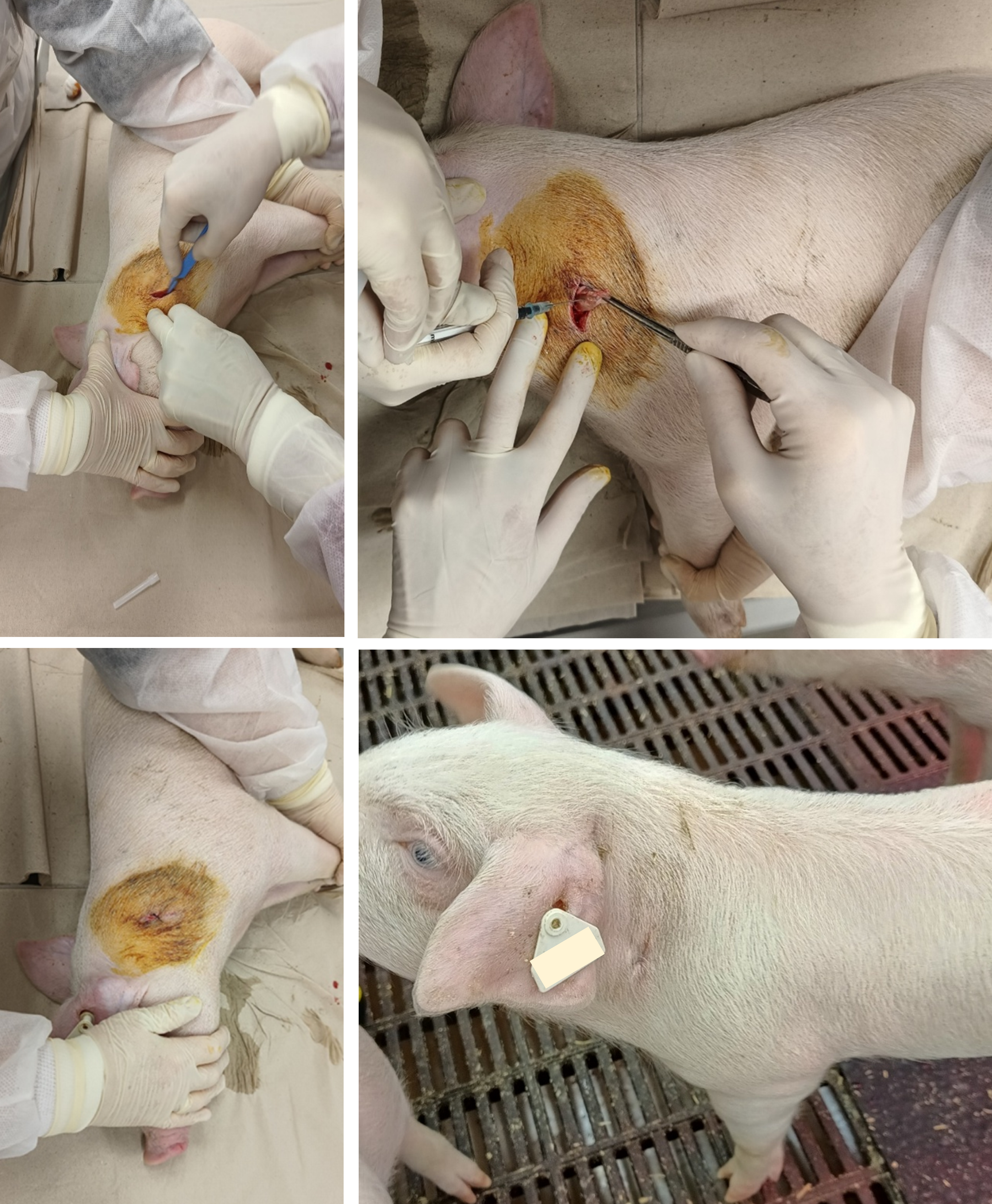

Supplement: Supplementary file 2 — Additional file 2. Surgery to inject S. suis in the left cervical lymph node. [file 13567_2025_1616_MOESM2_ESM.tif]

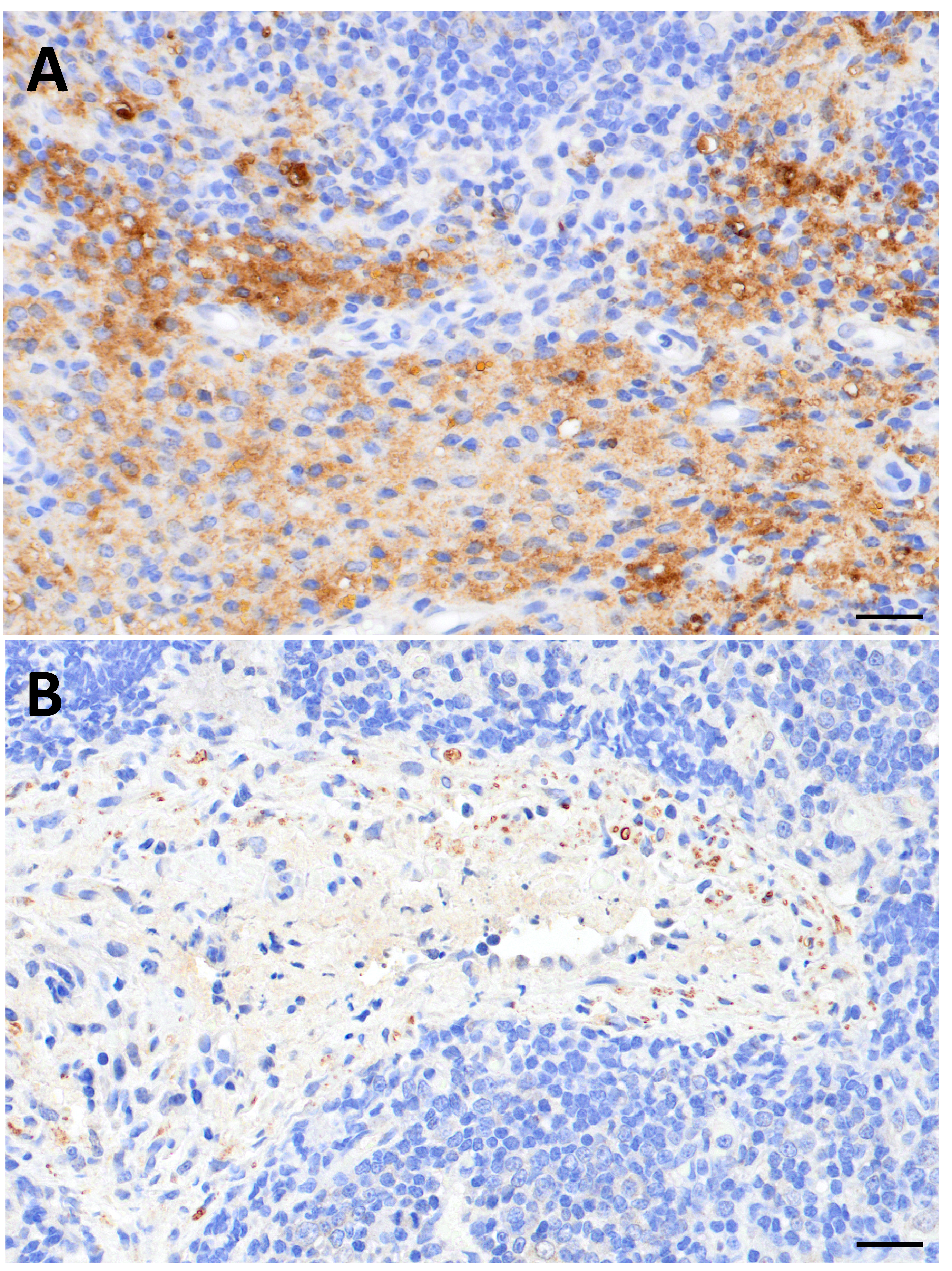

Supplement: Supplementary file 7 — Additional file 7. Distribution of S. suis-antigen within the left cervical lymph node. A Diffuse antigen within the sinusoids and paracortex. B Focal antigen within the sinusoids and paracortex. Serial sections from the left cervical lymph node were immunolabelled for S. suis antigen. Avidin-biotin-peroxidase complex method with diaminobenzidine as chomogen (brown) and haematoxylin counter-stain (blue); Nomarski differential interference contrast; scale bars = 20 µm. [file 13567_2025_1616_MOESM7_ESM.tif]
