# Supplementary material for: A new lymph node infection model for Streptococcus suis serotype 2 in pigs
Source: Vet Res. 2025 Oct 2;56:186. doi: 10.1186/s13567-025-01616-7 (PMC12490033; doi:10.1186/s13567-025-01616-7)
Supplement: Supplementary file 3 — Additional file 3. Reisolation of the infection strain from inner organs or joint fluid. [file 13567_2025_1616_MOESM3_ESM.pdf]

Additional file 3 Reisolation of the infection strain from inner organs or joint fluid

		Reisolation from											
	**No reisolation from inner organs/joints**	**1 site**	**2 sites**	**3 or more sites**	**Spleen**	**Lung**	**Liver**	**Liquor**	**Brain**	**Left heart valve**	**Blood**	**Serosa**	**Joint fluid**
**Non-infected**	2/2	0/2	0/2	0/2	0/2	0/2	0/2	0/2	0/2	0/2	0/2	0/2	0/2
**Infected (total)**	1/8	2/8	0/2	5/8	5/8	3/8	4/8	1/8	3/8	4/8	5/8	3/8	4/8
• **Group 1**	1/4	1/4	0/2	2/4	3/4	2/4	2/4	0/4	0/4	2/4	2/4	2/4	2/4
• **Group 2**	0/4	1/4	0/2	3/4	2/4	1/4	2/4	1/4	3/4	2/4	3/4	1/4	2/4
