# Supplementary material for: A new lymph node infection model for Streptococcus suis serotype 2 in pigs
Source: Vet Res. 2025 Oct 2;56:186. doi: 10.1186/s13567-025-01616-7 (PMC12490033; doi:10.1186/s13567-025-01616-7)
Supplement: Supplementary file 4 — Additional file 4. Reisolation of the infection strain from lymph nodes. [file 13567_2025_1616_MOESM4_ESM.pdf]

Additional file 4 Reisolation of the infection strain from lymph nodes

	No reisolation	Reisolation from 1 LN	From 2 LN	From 3 or more LN	*Ln. tracheo-bronchialis*	LN of the small intestine	*Ln. subiliacus* right	*Ln. subiliacus* left
**Non-infected**	2/2	0	0	0	0	0	0	0
**Infected (total)**	0/8	1/8	1/8	6/8	4/8	1/8	2/8	0/8
• **Group 1**	0/4	0/4	1/4	3/4	2/4	1/4	1/4	0/4
• **Group 2**	0/4	1/4	0/4	3/4	2/4	0/4	1/4	0/4

	Ln. popliteus right	*Ln. popliteus* left	*Ln. cervicalis* right	*Ln. cervicalis* left	*Ln. mandibularis* right	*Ln. mandibularis* left	*Ln. inguinalis* right	*Ln. inguinalis* left
**Non-infected**	0	0	0	0	0	0	0	0
**Infected (total)**	1/8	1/8	2/8	8/8	4/8	6/8	2/8	2/8
• **Group 1**	0/4	0/4	3/4	4/4	3/4	4/4	1/4	1/4
• **Group 2**	1/4	1/4	0/4	4/4	1/4	2/4	1/4	1/4
