# Supplementary material for: A new lymph node infection model for Streptococcus suis serotype 2 in pigs
Source: Vet Res. 2025 Oct 2;56:186. doi: 10.1186/s13567-025-01616-7 (PMC12490033; doi:10.1186/s13567-025-01616-7)
Supplement: Supplementary file 5 — Additional file 5. Detailed information to the individual pigs. [file 13567_2025_1616_MOESM5_ESM.pdf]

Additional file 5: Detailed information to the individual pigs

				Isolation from													
**Animal #**	**Time of euthanasia post inf.**	**Max. body temperature (°C)**	**Number of reisolation sites[a]**	**Spleen**	**Lung**	**Liver**	**Liquor**	**Brain**	**Left heart valve**	**Blood**	**Serosa[b]**	**Carpal joint left**	**Carpal joint right**	**Tarsal joint left**	**Tarsal joint right**	**Clinical signs (CNS)**	**Clinical signs (lameness)**
**1**	24 h	39.0	0														
**2**	70 h	39.1	0														
**3**	24 h	40.7	13	X	X	X			X	X	X	X			X		
**4**	24 h	41.1	18	X	X	X			X	X	X		X	X	X		hgr[c] l TJ [d]
**5**	24 h	39.1	2														
**6**	24 h	40.1	6	X													
**7**	48 h	41.3	8					X		X					X		hgr r TJ
**8**	48 h	40.8	2			X											hgr r CJ
**9**	56 h	41.1	11	X				X	X	X	X				X		hgr r TJ
**10**	70 h	41.0	10	X	X	X	X	X	X	X						X	lgr r TJ

[a] Isolation from lymph nodes, joint fluid, or inner organs
[b] Pleura, pericard, or peritoneum
[c] hgr – high grade/severe lameness; lgr – low grade/mild lameness
[d] l TJ – left tarsal joint; r TJ – right tarsal joint; r CJ – right carpal joint

Continuing Additional file 5: Detailed information to the individual pigs

Animal #	Isolation from											
	Ln. tracheo bronchialis	**LN of the small intestine**	***Ln. subiliacus right***	***Ln. subiliacus left***	***Ln. popliteus right***	***Ln. popliteus left***	***Ln. cervicalis right***	***Ln. cervicalis left***	***Ln. mandibularis right***	***Ln. mandibularis left***	***Ln. inguinalis right***	***Ln. inguinalis left***
**1**												
**2**												
**3**							X	X	X	X		X
**4**	X	X	X				X	X	X	X	X	
**5**								X		X		
**6**	X						X	X	X	X		
**7**					X			X	X	X	X	
**8**								X				
**9**	X		X			X		X				X
**10**	X							X		X		
