# Supplementary material for: A new lymph node infection model for Streptococcus suis serotype 2 in pigs
Source: Vet Res. 2025 Oct 2;56:186. doi: 10.1186/s13567-025-01616-7 (PMC12490033; doi:10.1186/s13567-025-01616-7)
Supplement: Supplementary file 6 — Additional file 6. Scoring of fibrinosuppurative lesions of piglets infected with 3 x 10^7^ CFU of S. suis strain 10. [file 13567_2025_1616_MOESM6_ESM.pdf]

Additional file 6: Scoring of fibrinosuppurative lesions of piglets infected with 3 x 10^7 CFU of *S. suis* strain 10

	pigs w/o lesions[a]	pigs with lesions in two or more locations[a]	**brain**			**serosae**			**joint**			**spleen and liver**			**lung**			**heart**		
			meningitis, chorioiditis			pleuritis or peritonitis			synovialitis			splenitis[b] or hepatitis			pneumonia			endocarditis		
			5[c]	3[d]	1[e]	5[c]	3[d]	1[e]	5[c]	4[c]	1[e]	5[c]	3[d]	1[e]	5[c]	3[d]	1[e]	5[c]	3[d]	1[e]
**Non-infected**	2/2	0/2	0/2	0/2	0/2	0/2	0/2	0/2	0/2	0/2	0/2	0/2	0/2	0/2	0/2	0/2	0/2	0/2	0/2	0/2
**Infected**	3/8	0/8	0/8	0/8	0/8	0/8	0/8	2/8	1/8	2/8	0/8	0/8	0/8	0/8	0/8	0/8	0/8	0/8	0/8	0/8
• **Group 1**	2/4	0/4	0/4	0/4	0/4	0/4	0/4	1/4	0/4	1/4	0/4	0/4	0/4	0/4	0/4	0/4	0/4	0/4	0/4	0/4
• **Group 2**[f]	1/4	0/4	0/4	0/4	0/4	0/4	0/4	1/4	1/4	1/4	0/4	0/4	0/4	0/4	0/4	0/4	0/4	0/4	0/4	0/4

[a] Only fibrinosuppurative lesions are considered. Individual single lymphohistiocytic infiltration are not scored.

[b] Neutrophilic accumulation of the splenic red pulp.

[c] Scoring of 4 and 5 indicates moderate to severe diffuse or oligo-/multifocal fibrinosuppurative inflammations.

[d] Scoring of 3 indicates mild multifocal fibrinosuppurative inflammation.

[e] Scoring of 1 and 2 indicates mild focal lymphohistiocytic or fibrinosuppurative inflammation.

[f] Using Martius-Scarlet-Blue stained sections, focal to multifocal fibrillar red masses were detected in the area of meningeal or plexus-related hemorrhages in the three animals with cultural detection of the *S. suis* challenge strain in the brain (Additional file 3). These red fibrils suggest intravital polymerization of fibrin and are interpreted as a very early stage of a fibrinous inflammation. These findings are not included in the score in line with our previous studies.
