# Supplementary material for: A new lymph node infection model for Streptococcus suis serotype 2 in pigs
Source: Vet Res. 2025 Oct 2;56:186. doi: 10.1186/s13567-025-01616-7 (PMC12490033; doi:10.1186/s13567-025-01616-7)
Supplement: Supplementary file 8 — Additional file 8. Histomorphological lesions in Ln. cervicalis superficialis dorsalis. [file 13567_2025_1616_MOESM8_ESM.pdf]

Additional file 7: Histomorphological lesions in Ln. cervicalis superficialis dorsalis

Pig[e] #	Left LN				Right LN			
	Follicular hyperplasia	Paracortical hyperplasia	Neutrophilic infiltration	Purulent lymphadenitis	Follicular hyperplasia	Paracortical hyperplasia	Neutrophilic infiltration	Purulent lymphadenitis
1	0[a]	0	1[b]	0	0	0	0	0
2	0	0	2[c]	0	0	0	2	2
3	0	0	2	1	0	0	1	1
4	0	0	2	1	0	0	1	1
5	0	0	1	0	ni[d]	0	0	0
6	0	0	1	0	0	0	1	1
7	0	0	2	1	0	0	1	1
8	0	0	1	0	0	0	1	0
9	0	0	1	0	0	0	1	1
10	0	0	1	0	ni	ni	ni	ni

[a]0 – no lesions detected

[b]1 – mild lesions detected

[c]2 – moderate lesions detected

[d]ni – not investigated

[e]pig #1,2: control group; pig #3-6: infected group 1; pig #7-10: infected group 2.
